# An Algorithm Measuring Donor Cell-Free DNA in Plasma of Cellular and Solid Organ Transplant Recipients That Does Not Require Donor or Recipient Genotyping

**DOI:** 10.3389/fcvm.2016.00033

**Published:** 2016-09-22

**Authors:** Paul M. K. Gordon, Aneal Khan, Umair Sajid, Nicholas Chang, Varun Suresh, Leo Dimnik, Ryan E. Lamont, Jillian S. Parboosingh, Steven R. Martin, Richard T. Pon, Jene Weatherhead, Shelly Wegener, Debra Isaac, Steven C. Greenway

**Affiliations:** ^1^Alberta Children’s Hospital Research Institute, University of Calgary, Calgary, AB, Canada; ^2^Department of Paediatrics, University of Calgary, Calgary, AB, Canada; ^3^Department of Medical Genetics, University of Calgary, Calgary, AB, Canada; ^4^Molecular Diagnostic Laboratory, Genetic Laboratory Services-South, Alberta Health Services, Calgary, AB, Canada; ^5^Department of Biochemistry and Molecular Biology, University of Calgary, Calgary, AB, Canada; ^6^Department of Cardiac Sciences, Libin Cardiovascular Institute of Alberta, University of Calgary, Calgary, AB, Canada

**Keywords:** cell-free DNA, transplantation, hepatocyte transplantation, heart, solid organ transplantation, biomarker

## Abstract

Cell-free DNA (cfDNA) has significant potential in the diagnosis and monitoring of clinical conditions. However, accurately and easily distinguishing the relative proportion of DNA molecules in a mixture derived from two different sources (i.e., donor and recipient tissues after transplantation) is challenging. In human cellular transplantation, there is currently no useable method to detect *in vivo* engraftment, and blood-based non-invasive tests for allograft rejection in solid organ transplantation are either non-specific or absent. Elevated levels of donor cfDNA have been shown to correlate with solid organ rejection, but complex methodology limits implementation of this promising biomarker. We describe a cost-effective method to quantify donor cfDNA in recipient plasma using a panel of high-frequency single nucleotide polymorphisms, next-generation (semiconductor) sequencing, and a novel mixture model algorithm. *In vitro*, our method accurately and rapidly determined donor:recipient DNA admixture. For *in vivo* testing, donor cfDNA was serially quantified in an infant with a urea cycle disorder after receiving six daily infusions of donor liver cells. Donor cfDNA isolated from 1 to 2 ml of recipient plasma was detected as late as 24 weeks after infusion suggesting engraftment. The percentage of circulating donor cfDNA was also assessed in pediatric and adult heart transplant recipients undergoing routine endomyocardial biopsy with levels observed to be stable over time and generally measuring <1% in cases without moderate or severe cellular rejection. Unlike existing non-invasive methods used to define the proportion of donor cfDNA in solid organ transplant patients, our assay does not require sex mismatch, donor genotyping, or whole-genome sequencing and potentially has broad application to detect cellular engraftment or allograft injury after transplantation.

## Introduction

The diagnosis of allograft rejection prompts an immediate change in management and is strongly associated with adverse outcomes. The gold standard for the detection of solid organ transplant (SOT) rejection requires tissue biopsy and histologic evaluation. However, this approach has significant limitations including the logistics of organizing the biopsy at short notice and risks associated with an invasive procedure. Non-invasive tests are safer and allow for more frequent monitoring, but current tests are generally too non-specific or insensitive for reliable rejection monitoring (1).

Allogeneic hepatocyte transplantation is emerging as a successful strategy to bridge patients to liver transplant in metabolic diseases (2). This approach is particularly useful in small infants where liver transplantation is not immediately feasible due to size and increased risk of technical complications. The principal assay to measure engraftment in liver cell transplantation (e.g. for urea cycle disorders) measures the conversion of ^13^C-acetate to ^13^C-urea (3), but this does not directly assess whether healthy donor cells are present. There is currently no acceptable, non-invasive method for the *in vivo* detection of donor cells that is useable in humans.

Cell-free DNA (cfDNA) is released primarily during cellular apoptosis and is found in the blood of all individuals (4–6). In the transplanted patient, cfDNA is derived from both recipient tissues and the donated organ or cells. Methodologies have been developed to quantify levels of donor-derived cfDNA in SOT with elevated levels associated with allograft injury from rejection or infection (7–14). Although promising, these studies required *a priori* knowledge of donor and recipient genotypes, donor–recipient sex mismatch, use of whole-genome sequencing (WGS), or the targeted amplification or sequencing of proprietary polymorphic markers or genomic regions. These issues represent significant barriers to the widespread use of cfDNA as a biomarker in transplantation.

We have developed an approach that does not require separate donor and recipient genotyping or WGS and can be completed rapidly using small volumes of plasma collected from the cellular or SOT recipient. We demonstrate our validation of this assay *in vitro* and its utility in the first application of cfDNA to measure the presence of donor cells in a human liver cell transplant recipient and for the quantification of donor-derived cfDNA in pediatric and adult heart transplant (HT) recipients.

## Materials and Methods

### Patient Information

This study was approved by the Conjoint Health Research Ethics Board at The University of Calgary (study ID REB14-1244). There were two distinct clinical patient scenarios. For the first application, the patient was a male infant with X-linked ornithine transcarbamylase deficiency (OTC) due to a deletion of exons 1–10 of the OTC gene. His peak ammonia in the first week of life was 1,368 μmol/l (normal <50 μmol/l). At 6 months of age, he received donor hepatocytes (0.3 × 10^9^ viable human liver cells per kilogram) from a single source *via* a portal venous catheter for six consecutive days using a protocol identical to the CCD05 trial (Cytonet GmbH & Co., KG, Weinheim, Germany). Approximately 20 days after the completion of the liver cell infusions, his ammonia had normalized to less than 50 μmol/l. Peripheral blood was drawn prior to the infusion of the donor liver cells and at 1 week (1 day after the 6th infusion), 1, 3, and 6 months post-infusion. The patient was ultimately bridged to a successful liver transplant at 16 months of age.

The second application involved blood collection from pediatric and adult HT recipients at the time of a clinically indicated endomyocardial biopsy (EMB). In children, EMB was performed at 1, 6, and 12 months post-HT and then annually up to 5 years. In adults, EMB was performed weekly for 1 month starting at 2 weeks post-HT then every 2–4 weeks until 6 months and then at 9, 12, 18, and 24 months. Five tissue samples were obtained from the right ventricular surface of the septum, stained with hematoxylin and eosin, and evaluated for acute cellular rejection (ACR) using light microscopy. Tissue samples were also evaluated for the presence of antibody-mediated rejection (AMR) by C4d-positive immunohistochemical staining. Biopsy findings of ACR or AMR were reported by the responsible pathologist (not involved in the study) using standard criteria (15, 16). Non-transplant controls were healthy volunteers.

### Isolation of Plasma Cell-Free DNA

After obtaining informed consent, whole blood (2–10 ml) was collected from patients in cfDNA blood collection tubes (Streck). Blood was centrifuged at either 4,000 rpm (larger volumes) or 13,000 rpm (smaller volumes) at 4°C for 15 min, the plasma removed, and centrifuged again at 13,000 rpm at 4°C for 15 min and the supernatant frozen at −80°C until used. Cell-free DNA was isolated from 1 to 4 ml of thawed plasma using the QIAamp Circulating Nucleic Acid kit (Qiagen). Isolated cfDNA was quantified using a Qubit fluorometer (Life Technologies), and purity (absence of nuclear DNA) was confirmed using a TapeStation 2200 D1000 tape (Agilent). In the case of contaminating genomic DNA, a size selection step was incorporated into our protocol to isolate fragmented cfDNA of 100–300 bp using magnetic SPRIselect or AMPure XP beads (Beckman Coulter).

### Library Preparation and Sequencing

Barcoded sequencing libraries were prepared using size-selected cfDNA (1 ng) and the HID-Ion Ampliseq Identity Panel and Ampliseq Library kits (ThermoFisher Scientific). The HID-Ion Ampliseq Identity Panel that we used contained 124 SNPs on the autosomal and Y chromosomes curated from the literature (17–19) and designed for forensic analyses from degraded samples. Libraries were quantified using Qubit and TapeStation assays, diluted to 20 pM, pooled, and then put on the Ion Torrent OneTouch 2 system (ThermoFisher Scientific) for the creation of template beads. Beads were loaded onto an Ion 316 or 318 semiconductor chip for sequencing on the Ion Torrent PGM platform (ThermoFisher Scientific) in 200 bp mode. The original equipment manufacturer (OEM) base caller with default parameters was used, and resultant sequences were mapped to the GRCh37 reference sequence using the OEM tmap software in stage1/map4 mode.

### Modeling Donor–Recipient cfDNA Admixture

**Figure 1:**
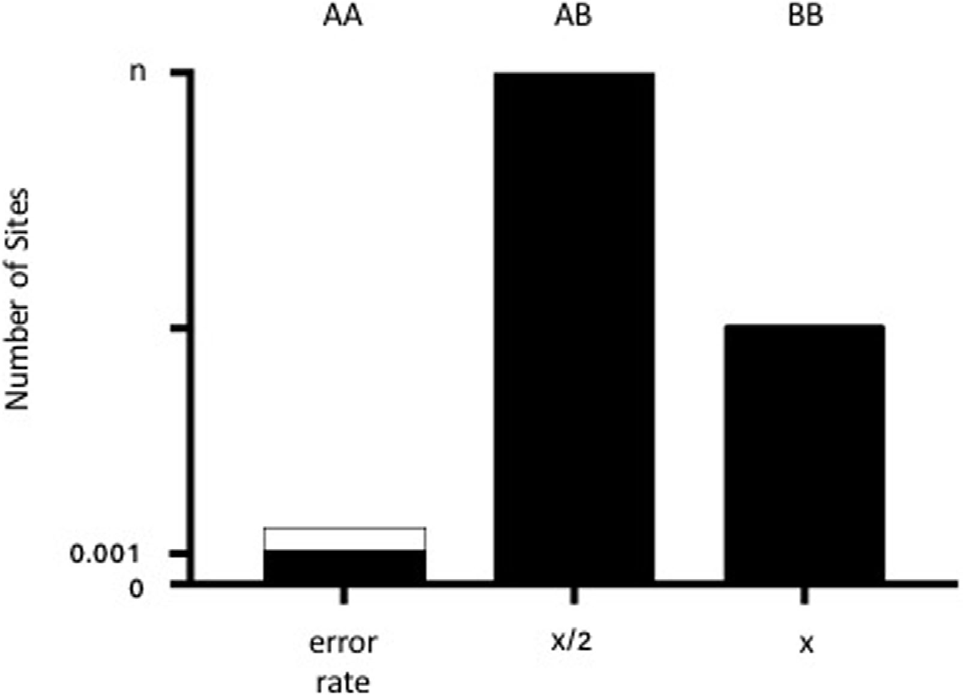
**Diagram of the admixture theory underlying our algorithm**. When donor and recipient alleles are identical (AA), the signal represents the theoretical error rate for base calling (0.001 or 0.1% from the BAQ score of 30). The dotted lines demonstrate the pooled estimate of error rates across samples. When there is one shared allele (AB), then the % donor (*x*) is equal to *x*/2 for *n* number of sites (where *n* generally equals 42). When there are no shared alleles (BB), then the number of SNPs is equal to *x*.

Biallelic autosomal loci from the Human Identity Panel homozygous in the major (“recipient”) sample contributor were selected. A locus was considered homozygous if the variant was present in >86% of the sequence reads with base quality scores ≥30. Loci with >5% ambiguous base calls were excluded. Non-consensus data (minor base calls) at recipient homozygous loci were then collectively used to estimate the minor (“donor”) contribution through a statistical procedure known as Finite Mixture Modeling with linear constraints. For a human sample with two contributors, biology dictates that the donor model should have three non-consensus signals: (1) both alleles same as recipient 25% of the time, (2) one allele same as recipient 50% of the time, and (3) neither allele same as recipient 25% of the time (Figure 1). The “normalmixEM” method from the open source *R* package “mixtools” was used to generate the models (20). Mixture modeling requires simulation and, therefore, can yield different results for the same input data on different software runs. The mean and SD of 10,000 simulations were taken as the final result, and the overall calculation was completed in few minutes. Using a base alignment quality (BAQ) score (Phred-like score) of Q30 gives us a theoretical error rate of 0.001. A pooled estimate of error rate was calculated using all samples run on the same chip.

Biologically relevant constraints were added to improve the estimates and yield consistent results between runs. Since the panel consists of independent evenly biallelic loci, a 1:2:1 intensity ratio for the three donor cfDNA signals was used, i.e., same alleles as recipient (signal = error rate), one shared allele (OSA), and no shared alleles (NSA). An initial estimate of 0.1% for the mean of the error rate was provided, based on the base quality cutoff (BAQ score of 30 = 0.1% error rate). A 1:2 ratio constraint for the means of the OSA and NSA was enforced. A 1:2 constraint for the OSA:NSA SDs was also employed. For each simulation, the final estimate of donor cfDNA contribution consists of the NSA%, minus the final error estimate. For our data, the Gaussian (“normal”) distribution yielded more accurate results than the gamma distribution or other methods available in the mixtools package.

To test our algorithm, HapMap DNA samples (Coriell Institute for Medical Research) were used to simulate a mixture of two separate populations as previously described (9) with sample NA07348 designated as the “recipient” and NA10830 as the “donor.” Six mixtures (total of 1 μg for each mixture) were created with increasing concentrations of NA10830 (0, 0.5, 1, 2, 4, and 8%). Concentrations were chosen based on data suggesting that donor cfDNA is present at baseline between 0 and 1% and increases with rejection to at least 3–6% in heart transplantation (9). We prepared libraries of these six genomic (gDNA) mixtures in triplicate and sequenced them as described above.

### Statistical Analysis

Data are shown as means ± SD. Calculations were performed in Excel (Microsoft Corporation) for calculation of the linear correlation between the expected and measured donor gDNA concentrations, and the Student’s *t*-test was used to determine if there was a significant difference (defined as *p* < 0.05) between sample groups.

## Results

**Figure 2:**
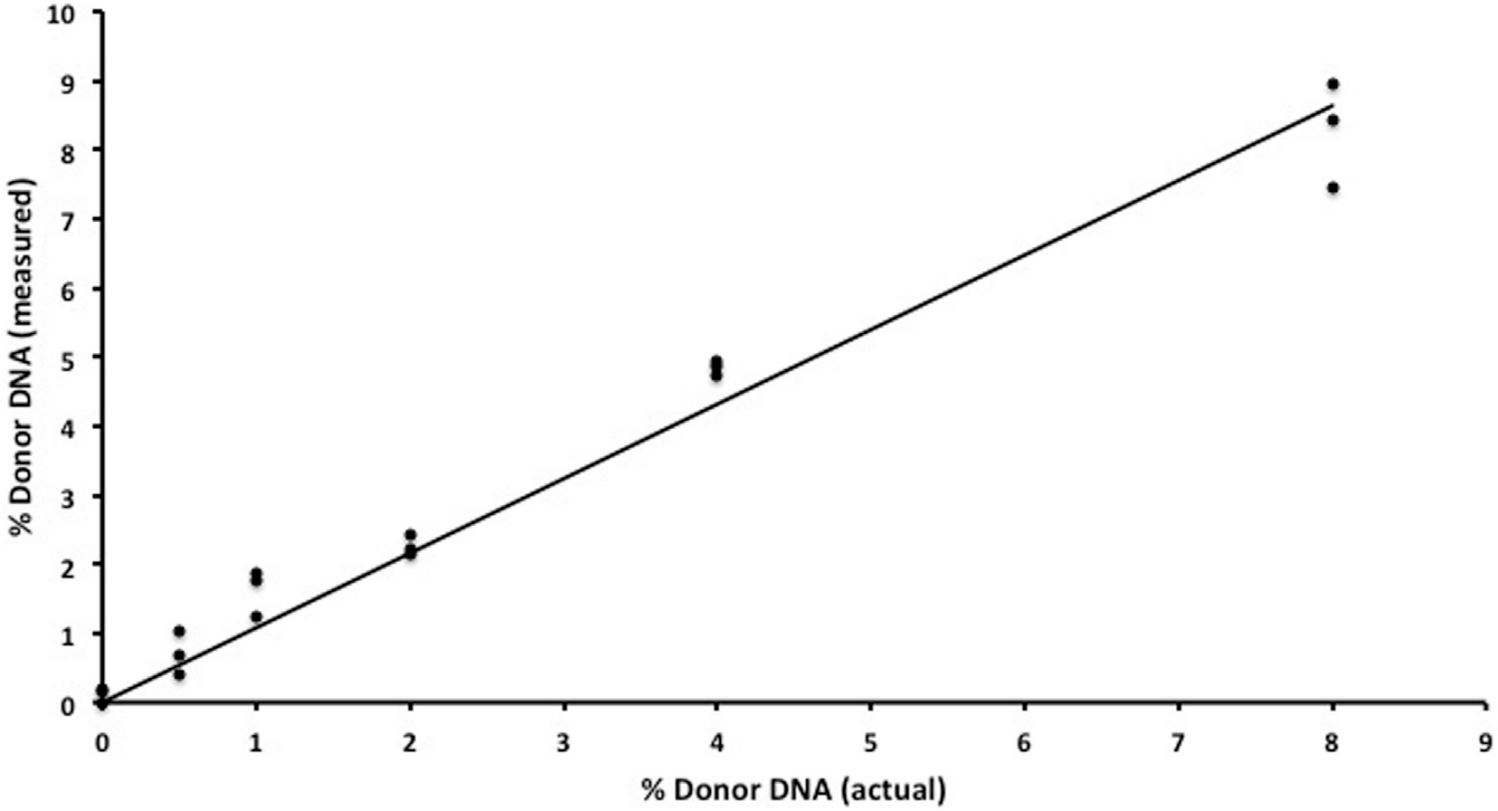
***In vitro* assay validation**. Results for semiconductor sequencing of six separate genomic DNA mixtures (0, 0.5, 1, 2, 4, and 8%) with strong correlation (*R*^2^ = 0.97) between the expected (actual) and observed (measured) levels of donor cfDNA as calculated by our algorithm. The experiment was performed in triplicate with each individual admixture result plotted.

We began by replicating previous work (9) using two gDNA samples and WGS (SOLiD platform, Applied Biosystems) to detect increasing amounts of one sample in a mixture. We were successful in replicating this part of the study from Snyder et al. (9) and found a strong (*R*^2^ = 0.98) correlation between the expected and measured donor DNA concentrations (data not shown) but were dismayed by the cost, inefficiency, and complex bioinformatics required for this assay. We then developed an alternative methodology that we tested using identical mixtures of HapMap gDNA. We were also able to accurately detect increasing amounts of an individual sample in a mixture and at a fraction of the cost of the WGS method (Table 1). There was strong (*R*^2^ = 0.97) zero-intercept linear correlation between the expected and measured donor DNA concentrations over three independent replicates (Figure 2).

**Table 1:** **Comparison of sequencing platforms for the measurement of donor cfDNA**.

Item	Ion Torrent	WGS (Illumina)
Sequencing overview	Ion 316 chip v2 (200 bp reads)	Paired end (2 × 150 bp)
Library overview	AmpliSeq Identity Panel (124 SNPs)	DNA fragment library (3 Gb genome)
Expected output	300–600 Mb	100–120 Gb
Expected reads	2–3 million	800 million
Coverage per SNP	≥1000	≥30
Cost of library preparation	$255	$85
Sequencing cost	$700	$6670
Turnaround time	3 days	3 days
Samples per run	8	1
Total cost per sample	$163.13	$6755

*With the ability to accommodate multiple samples per sequencing run due to the lower required output, the Ion Torrent platform is more cost-effective compared to WGS*.

**Figure 3:**
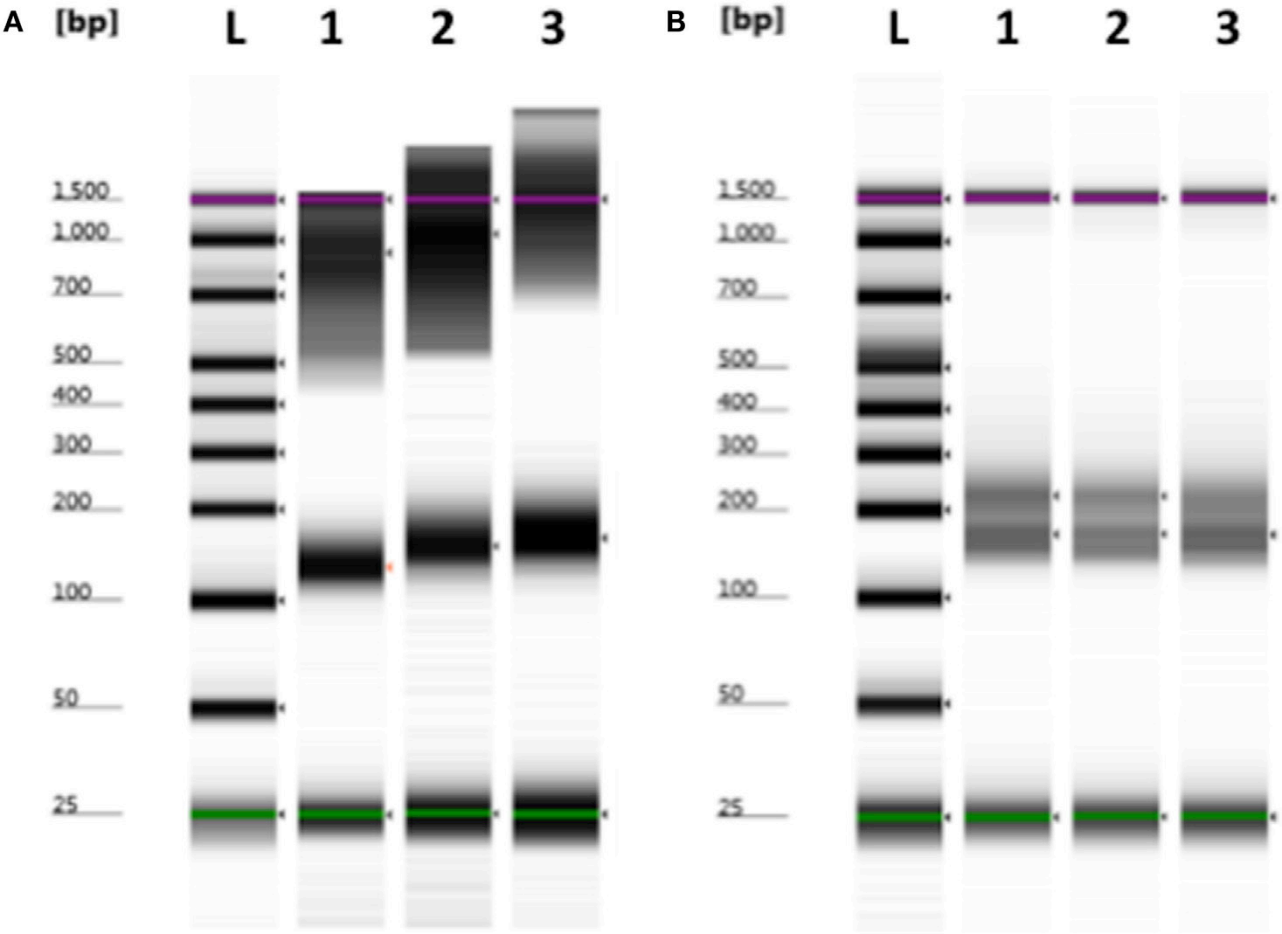
**Removal of contaminating nuclear DNA from plasma using size selection**. **(A)** Example of plasma cfDNA (100–200 bp) preparation containing larger fragments (>500 bp) of genomic DNA. **(B)** Contaminating gDNA was removed by incorporating a size-selection step prior to library construction. L, ladder; 1–3, plasma cfDNA samples. Green (minimum) and purple (maximum) lines indicate the sizing range of the assay.

**Figure 4:**
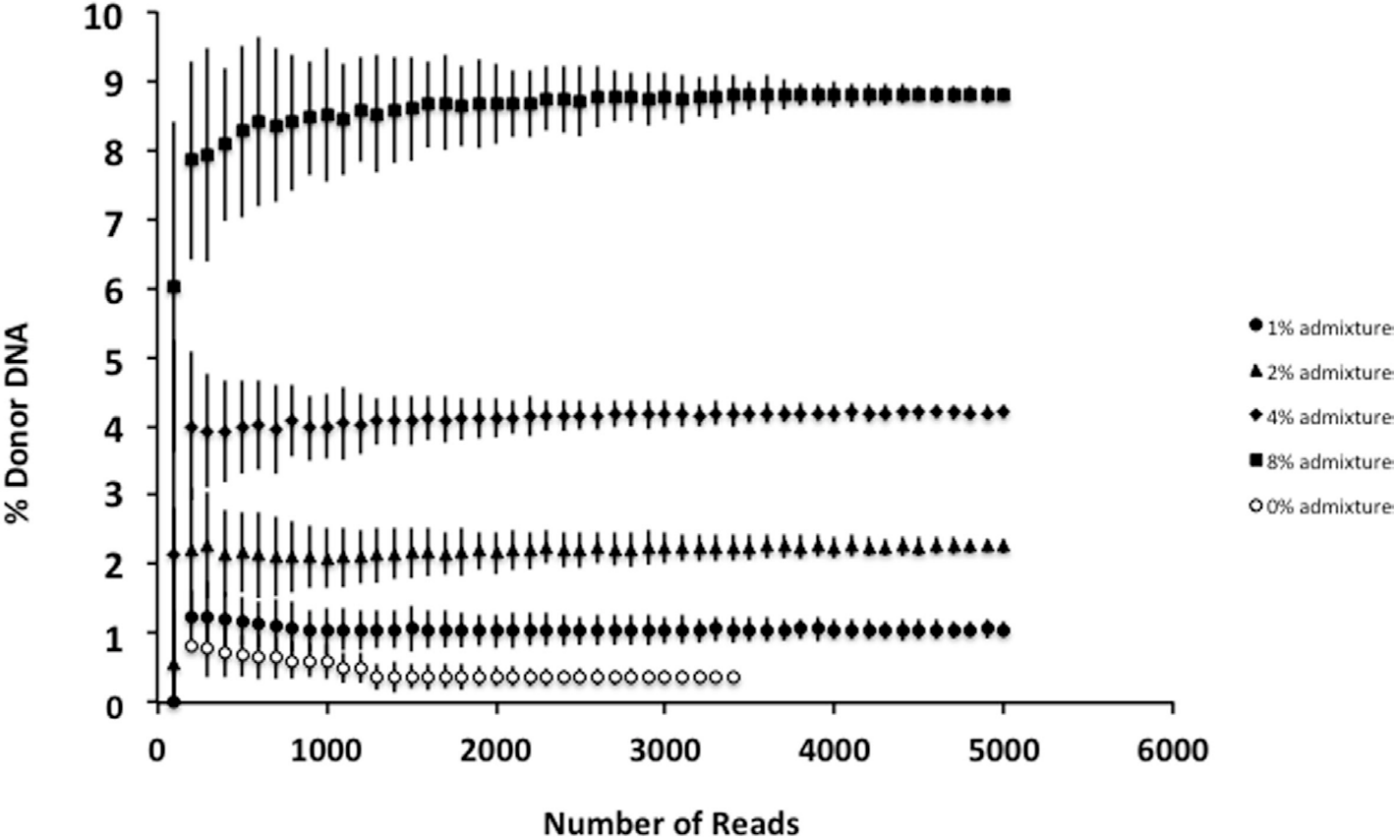
**Effect of increasing read depth on proportion estimates**. Using genomic DNA mixtures (0, 1, 2, 4, and 8%), we see that for an individual sample, read depth >1200–1500 is associated with reduced error estimate for the proportion of donor cfDNA present. Fewer reads are associated with decreased confidence in the estimate for the proportion of donor cfDNA. Data are based upon sequencing of individual admixtures performed in triplicate.

Although smaller blood volumes (2–5 ml) were often collected (due to small patient size and pediatric blood volumes) in Streck tubes designed for larger blood volumes (10 ml), we were able to successfully isolate cfDNA in all cases. Different centrifugation speeds for the separation of plasma from whole blood were tested with equivalence in cfDNA yield (data not shown). Of five kits tested, greatest and most reliable yields of cfDNA from plasma were obtained using the QIAamp Circulating Nucleic Acid kit (data not shown). In our initial experiments, contaminating gDNA complicated estimates of cfDNA yield (particularly with smaller volumes of blood), and we added a size-selection step (Figure 3) before proceeding onto library construction. This process was successfully used to isolate cfDNA from all patient plasma samples with sufficient material recovered to generate acceptable sequencing libraries. As shown in Figure 4, estimates of donor DNA plateaued at approximately 1200–1500 reads per base. Since approximately 300–600 Mb of sequencing reads can be obtained using the 316 PGM chip, we used up to eight individual samples per chip.

### Detection of Infused Donor Liver Cells

**Figure 5:**
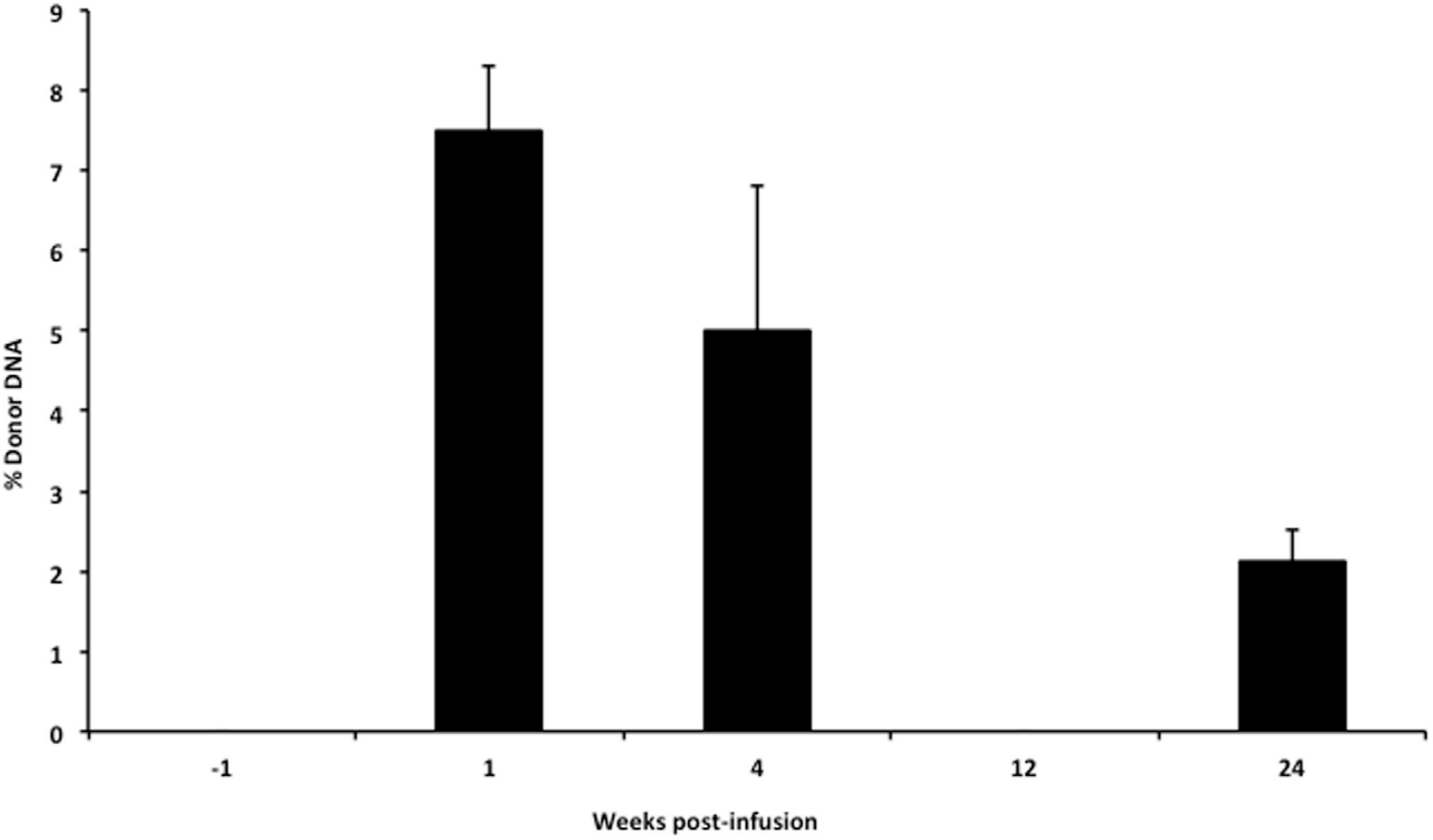
**Levels of donor cfDNA pre- and post-liver cell transplantation**. The percentage of donor cfDNA present in the blood of a single patient with X-linked ornithine transcarbamylase deficiency both before (−1 week) and at 1, 4, 12, and 24 weeks after the initiation of donor hepatocyte infusions. Donor cfDNA was not detectable by our assay pre-infusion or at 12 weeks post-transplant. The percentage of donor cfDNA is shown for each group ±SD.

In our infant with OTC deficiency, donor cfDNA was quantified prior to the infusion of donor hepatocytes and at serial time points post-infusion to provide a measure of engraftment success (Figure 5). There was a large initial surge in the amount of donor cfDNA detected at week 1 (1 day after the 6th liver cell infusion), at 1-month post-infusion, and then the levels steadily declined. At 3 months post-infusion, we failed to detect any donor cfDNA in the single sample that was collected (despite successful isolation of cfDNA from the plasma), but donor cfDNA was detectable in the subsequent sample at 6 months post-infusion.

### Detection of Donor cfDNA in Heart Transplantation

**Figure 6:**
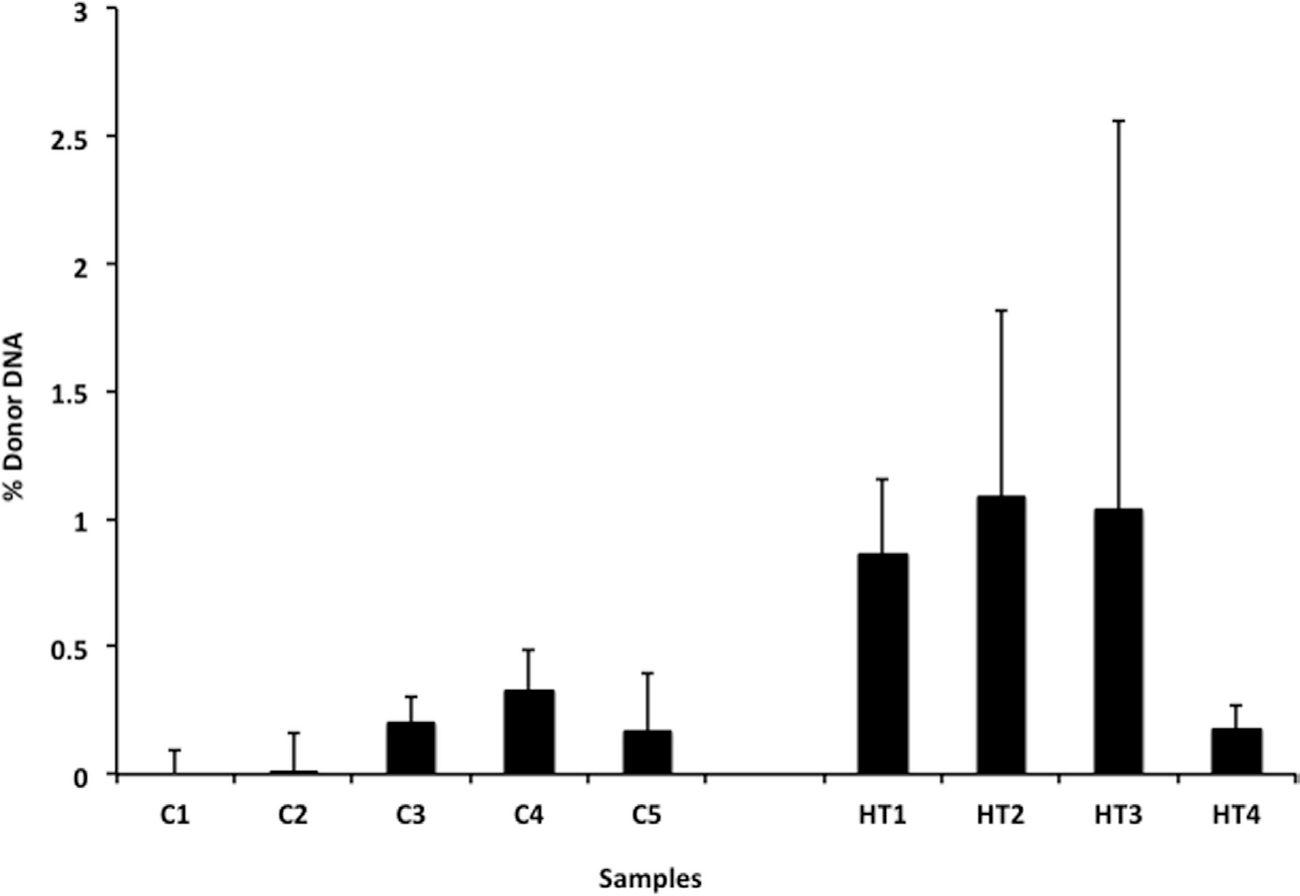
**Levels of donor cfDNA in plasma from healthy pediatric heart transplant patients**. Percentage of donor cfDNA present in non-transplant controls (C1–5, representing background noise or error) and in healthy pediatric heart transplant patients (HT1–4) free of rejection (ACR 0R and pAMR 0) or allograft dysfunction who underwent routine surveillance endomyocardial biopsies. The percentage of donor cfDNA is shown for each group ± SD. Comparing average levels for the two groups, the amount of donor cfDNA in transplant patients is significantly elevated above background controls (*p* = 0.02).

**Figure 7:**
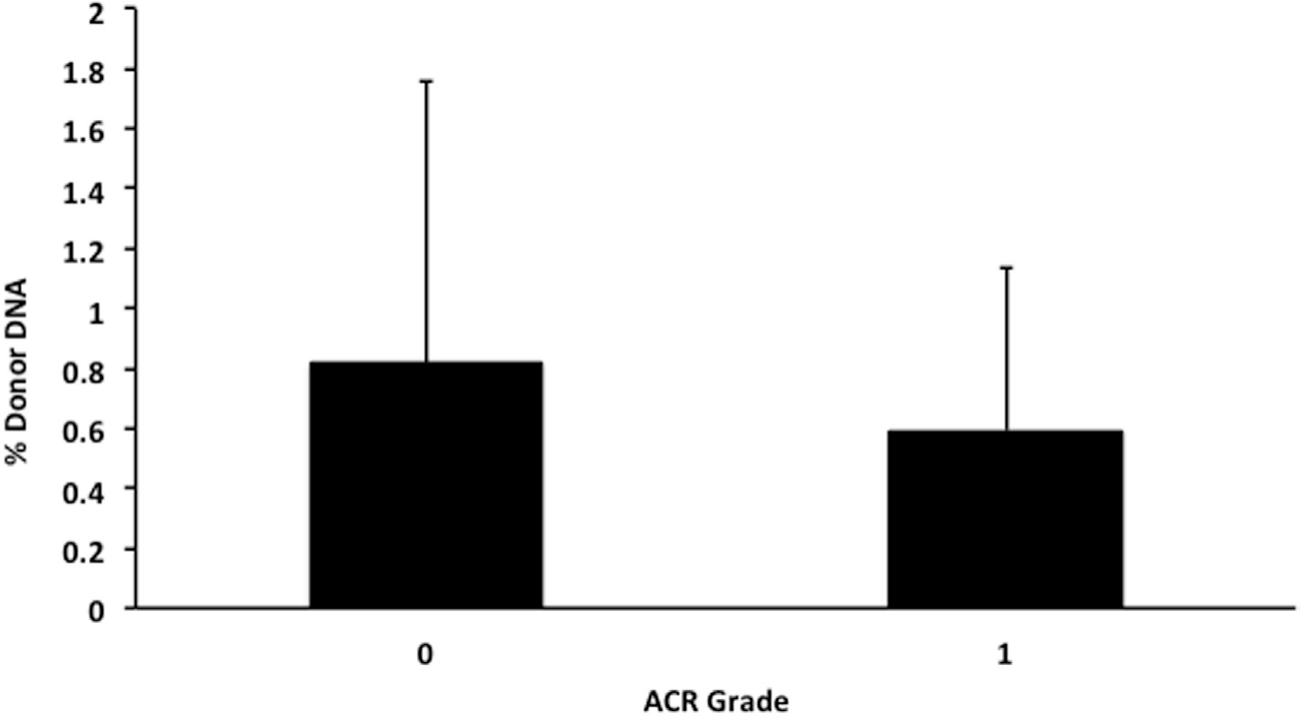
**Levels of donor cfDNA in plasma from adult heart transplant patients without significant rejection**. No significant difference (*p* = 0.7) was detected between levels of donor cfDNA from adult heart transplant recipients with none (ACR 0R, *n* = 15) or mild (ACR 1R, *n* = 24) cellular rejection on routine endomyocardial biopsy. The percentage of donor cfDNA is shown for each group ± SD.

**Figure 8:**
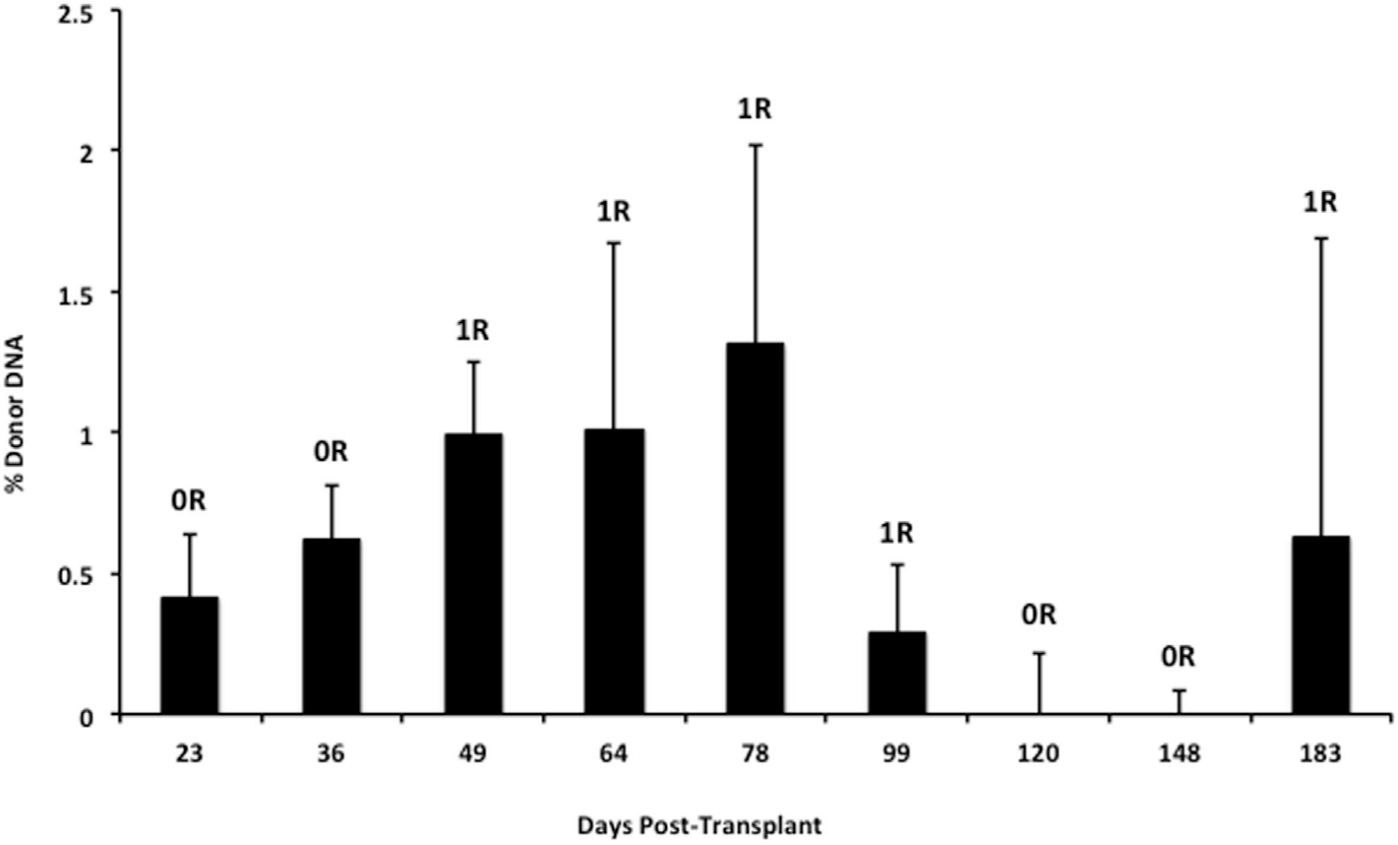
**Stable serial cfDNA levels post-transplantation for a single adult heart transplant recipient**. Samples were taken at the time of routine post-transplant surveillance endomyocardial biopsies from 23 to 183 days post-transplant, and all were free of serious rejection and reported as either no (0R) or mild (1R) ACR with no evidence of AMR. The percentage of donor cfDNA is shown for each time point with error estimates as SD.

We also applied our novel assay to plasma collected from healthy non-transplant controls (to estimate background in our assay due to the error rate for base calling) and from pediatric and adult HT recipients undergoing a clinically indicated EMB. Relatively low levels of plasma donor cfDNA were seen for all pediatric HT patients, as expected, since all patients were asymptomatic with no evidence of dysfunction by echocardiography, and none had evidence of significant ACR or AMR by EMB with all graded as ACR 0R and pAMR 0 according to ISHLT grading criteria (15, 16). However, as a group, the levels of donor cfDNA were significantly higher (*p* = 0.02 by Student’s *t*-test) than background control levels (Figure 6). In the samples collected from adult HT recipients, we did not find a statistically significant difference (*p* = 0.7) between pooled donor cfDNA levels in patients with ACR 0R (*n* = 15) or 1R (*n* = 24) and who were free of AMR (Figure 7). In serial samples collected from individual adult patients undergoing routine protocol EMB post-HT, we saw that levels of cfDNA generally remained low (<1%) and stable post-transplant (representative example shown in Figure 8).

## Discussion

We began by replicating previous work using gDNA and WGS to detect increasing amounts of one DNA sample in a mixture (9). Based on our experience, we developed an alternative methodology that used the Ion PGM semiconductor sequencing platform to sequence a commercially available panel of 124 highly polymorphic SNPs originally developed for the forensic identification of individual DNA samples. Relatively deep sequencing (at least 1200-fold coverage) of selected high-quality autosomal SNPs (on average 42 SNPs) combined with a novel and biologically relevant statistical model enabled us to non-invasively, rapidly, and accurately quantify the proportion of donor alleles from a small sample of recipient plasma.

The clinical benefit of liver cell infusion as a bridge to transplantation for metabolic liver disease has been established. However, a direct, non-invasive, and clinically useful method for the detection of donor cell engraftment in the recipient is not available. Our assay provided a direct measure of donor hepatocyte cfDNA, which correlated with improved ammonia control in this patient and, therefore, likely represented material from engrafted liver cells. In our patient, we saw a rapid increase in the percentage of donor cfDNA detected at 1-week post-infusion. This may represent the early death of donor cells that do not successfully engraft and is analogous to the elevated levels of donor cfDNA seen early post-heart or lung transplantation (7, 21). Levels of donor cfDNA had begun to decline by 1-month post-infusion, and at 3 months post-infusion, we did not detect any donor cfDNA. The lack of cfDNA at week 12 was a single measure and may be primarily due to sample collection that took place after >12 h of hemodiafiltration treating a temporary episode of hyperammonemia. Kohlova and colleagues demonstrated that cfDNA levels increased after hemodialysis (22), but these were stable renal failure patients undergoing routine hemodialysis, which likely would not have dialyzed cfDNA. In our patient, the hyperammonemia required aggressive hemodiafiltration, which can remove larger molecules (23) and could have removed the cfDNA. Alternatively, given that just a single sample was obtained, this could represent an error in our methodology. However, we obtained a similar result from repeat sequencing of the library. When sample collection was repeated at 24 weeks post-liver cell infusion, we ensured that >72 h had passed from the time of hemodiafiltration, and this sample indeed showed that donor liver cells were still present in the recipient. Therefore, the sustained detection of donor cfDNA at 4 and 24 weeks post-liver cell infusion in our patient receiving concomitant immunosuppression likely reflects sustained engraftment of donor liver cells. In animal studies, hepatocytes were found engrafted in the liver and spleen (24) within 1 week of liver cell infusion, and in humans with acute liver failure, engrafted liver cells were found at 14 days post-liver cell infusion by biopsy (25). In the future, examination of the recipient liver at the time of transplantation could allow this to be confirmed although the size of the recipient liver, and the relatively sparse number of donor cells makes this potentially difficult.

In our cohort of pediatric HT recipients, we demonstrated that low levels of donor cfDNA (generally <1%) can be detected in the absence of allograft dysfunction or rejection, but that these levels are distinct from non-transplant controls where the presence of “donor” cfDNA represents the error rate for base calling. Using our assay, we found that levels of donor cfDNA from adult HT recipients with either no (ACR 0R) or mild (ACR 1R) rejection do not differ significantly. This finding is consistent with the clinical practice of treating ACR 0R and 1R as equivalent and generally not altering management or increasing immunosuppression.

One concern regarding the use of donor cfDNA as a biomarker is that levels of cfDNA in an individual may fluctuate over time. Changes in total circulating cfDNA have been documented after extreme exercise, trauma, or infection (26–28) but, we found that, for a transplant patient who is clinically well, serial levels of donor cfDNA appear to be relatively stable. The proportion of donor cfDNA is likely to increase in other instances of allograft injury including cardiac allograft vasculopathy (11) and BK virus nephropathy (10), but further characterization of the donor cfDNA molecules released from the injured allograft may provide insight into the mechanism underlying allograft injury and may provide specificity for this sensitive biomarker. This may provide cfDNA with advantages not available for the non-specific circulating biomarkers currently used in kidney or liver transplantation.

Due to the numerous benefits a non-invasive test for rejection would provide many potential blood biomarkers have been tested but with little success (1). However, donor cfDNA is a promising new candidate and may more reliably reflect allograft health, since it is directly derived from the transplanted cells or organ (29). Unfortunately, quantification of donor cfDNA using WGS is limited by expense, the need for *a priori* genotyping and complex bioinformatics. Other methods to measure cfDNA are limited by the requirement for donor–recipient sex mismatch, independent donor and recipient genotyping or proprietary components (8, 10–12). Our assay facilitates potential clinical applicability by using a readily available panel of SNPs and an algorithm that removes the need for donor and recipient genotyping. Furthermore, since fewer reads are required to distinguish between donor and recipient molecules, we are able to use a less expensive platform, which can complete the required sequencing within 4 h. This rapidity enables our assay to potentially compete with the time required for routine clinical biopsy interpretation. Currently, an individual technician can prepare 8–16 sample libraries per day, and automation may increase this number. Although we used semiconductor sequencing, the principle of this assay and the algorithm can be used with any high-throughput platform, because noise estimation is independently performed for each sequencing run. Issues with sampling error related to the small number of molecules sampled may be mitigated by increasing panel size, an option not readily available for alternative, targeted approaches (8).

### Limitations

There are several limitations to our approach. Our assay does not assess the functional health of the engrafted cells and clinical correlation (e.g., levels of ammonium, need for dialysis) is still required. Similarly, information collected reflects only allograft engraftment/health, and we do not obtain additional information regarding the presence of circulating viruses, which may be diagnostically useful or helpful in estimating an individual’s degree of immunosuppression (21, 30). With our algorithm, there are several assumptions made. We are limited to situations where the donor fraction is <14%. However, based on published reports of allograft rejection in patients >14 days post-transplant demonstrating levels of donor-derived cfDNA generally <10% (7, 14), we feel that our assay still has utility in the detection of rejection in stable SOT patients who are further out from transplantation – arguably, the population where a non-invasive method to diagnose allograft dysfunction is most needed and useful. However, this may only be optimal for surveillance post-heart transplantation and may be inappropriate for other organs (e.g., lung and liver) where the percentage of circulating donor cfDNA can be substantially higher (21). These organ-specific differences may make optimization of our assay for individual organs/cells necessary. Furthermore, since our assay relies upon differences in minor allele frequency between individuals, our assay is likely not useful for closely related donor–recipient pairs as seen after living-related kidney donation. In our early experience, we found that measures of cfDNA quantity after isolation were frequently over-estimated related to the presence of nuclear genomic DNA from white blood cells contaminating our plasma preparations, and this led us to incorporate the size selection step. This extra procedure did result in the loss of some cfDNA (although presumably donor and recipient molecules are affected equally), and this may have contributed to some of the variability in our assay. The size-selection step may not be necessary if contamination of the plasma by the white blood cells in the buffy coat is avoided, but size selection may be useful when there are very small volumes of blood available (i.e., as obtained from neonates or small infants), and the likelihood of contamination is greater. Further efforts to improve the consistency of sample isolation and quantify the number of donor molecules per milliliter of plasma may improve reliability and accuracy of percent donor DNA estimates. Biological validation of our assay also remains limited at this time due to small patient numbers.

## Conclusion

We have described an assay that non-invasively, rapidly, and accurately quantifies the proportion of donor cfDNA in plasma from recipients of both cellular and SOTs. This assay is scalable and uses a simple algorithm that does not require donor:recipient sex mismatch or *a priori* genotyping. We have demonstrated the utility of our approach *in vitro* and in proof-of-principle for two *in vivo* clinical transplant applications; the detection of cellular engraftment and the non-invasive detection of rejection. Further validation in additional transplant recipients is required, and recruitment of pediatric and adult patients is ongoing.

## Author Contributions

PG and SG were responsible for study design. PG wrote the algorithm. AK and SM performed the clinical hepatocyte transplant. US, NC, and VS performed the work. LD, RL, JP, RP, JW, and SW performed and optimized the sequencing. DI, US, NC, and SG recruited the heart transplant patients. SG and PG performed the data analysis. SG wrote the manuscript. All authors reviewed the manuscript.

## Conflict of Interest Statement

This research was conducted in the absence of any commercial or financial relationships that could be construed as a potential conflict of interest. PG and SG have applied for a patent related to the described methodology.

## Abbreviations

ACR, acute cellular rejection; AMR, antibody-mediated rejection; BAQ, base alignment quality; cfDNA, cell-free DNA; EMB, endomyocardial biopsy; gDNA, genomic DNA; HT, heart transplant; NSA, no shared alleles; OEM, original equipment manufacturer; OSA, one shared allele; OTC, ornithine transcarbamylase deficiency; SD, standard deviation; SNPs, single nucleotide polymorphisms; SOT, solid organ transplant; WGS, whole-genome sequencing.
